# A Pilot Study on the Association of Internet Use with Sports Practice and Sex in Italian Adolescents

**DOI:** 10.3390/healthcare11233075

**Published:** 2023-11-30

**Authors:** Stefania Toselli, Alessia Grigoletto, Luciana Zaccagni, Emanuela Gualdi-Russo, Natascia Rinaldo

**Affiliations:** 1 Department for Life Quality Studies, University of Bologna, 47921 Rimini, Italy; stefania.toselli@unibo.it; 2 Department of Biomedical and Neuromotor Sciences, University of Bologna, 40126 Bologna, Italy; alessia.grigoletto2@unibo.it; 3 Department of Neuroscience and Rehabilitation, Faculty of Medicine, Pharmacy and Prevention, University of Ferrara, 44121 Ferrara, Italy; emanuela.gualdi@unife.it (E.G.-R.); natascia.rinaldo@unife.it (N.R.); 4 Center for Exercise Science and Sports, University of Ferrara, 44123 Ferrara, Italy

**Keywords:** adolescence, risk behaviors, technology/media/social media, gender

## Abstract

The use of the Internet has several positive functions, but at the same time it also represents a health risk, especially for adolescents. The increased use of the Internet in recent decades has also been linked to an increase in problematic Internet use, which has now become a global social issue. During the COVID-19 pandemic, Internet use increased even more and influenced adolescents’ habits, leading to an increase in sedentary behaviors. The aim of this study was to understand whether Internet addiction differed between sportive and nonsportive adolescents and between males and females. We analyzed two samples of adolescents, with different behaviors regarding sports practice. Internet addiction was assessed by the administration of a validated questionnaire (UADI) via an online survey. Although all the mean values of both the subgroups fell into the “non-pathological” category, differences between sports groups and sexes were found, with different trends for each one of the five dimensions of Internet use. In conclusion, sportive adolescents generally showed less severe Internet addiction; therefore, encouraging sports practice can help to fight wrong habits related to a sedentary lifestyle. The promotion of sports practice in these ages is therefore a priority aspect, especially in females, who showed a general predisposition to escape from reality in comparison to males.

## 1. Introduction

The Internet has remarkably positive functions, including its use as a supplementary instrument adaptive to reality, and effective for self-realization, experimentation, and research. Its use has offered new opportunities and challenges, allowing connections with other people without geographical or time constraints [1]. However, it also represents a risk to health, as it has the negative capability to induce addiction, especially in adolescents [2,3]. Addiction involves the loss of control over the use of a substance or over certain behaviors despite the knowledge of possible negative consequences. In recent years, Internet use has increased among young people and the COVID-19 health emergency has worsened it [4]. Among the array of online leisure activities, social networking is the most popular, as reflected in the escalation of the number of users over the past two decades [5]. Another highly popular online leisure activity is gaming [6]. Internet addiction or problematic Internet use (PIU) has emerged. Internet addiction is a type of behavioral addiction; the physical manifestations are compulsive Internet use, and the psychological manifestations are behavioral problems caused by withdrawal from Internet addiction. Internet addiction is defined as an inability to control online participation, which results in substantial impairment of individuals’ function in their various life domains for a prolonged time [7]. PIU is characterized by behaviors associated with poor control, continuous use, and cognitive concern regarding the Internet, which can carry a series of negative consequences in different areas of an individual’s life [8,9]. These addictive behaviors are, in turn, related to higher levels of psychological and physical health problems [10,11]. In fact, Internet addiction generally leads to psychological, social, educational, or vocational problems in a person’s life [12,13]. Internet-addicted people are more likely to have a disturbed mental state compared to the people without this problem at all levels of mental health. Internet addiction is often accompanied by such comorbidities as impulsiveness, depression, anxiety, and obsessive-compulsive disorders [14]. Adolescents’ aggressive behavior during COVID-19 was positively related to Internet addiction, anxiety and depression [15]. Furthermore, different studies indicate a relationship between PIU and use of substances such as alcohol and tobacco [16,17,18]. Internet use may increase the likelihood of cyber victimization, such as impersonation and social exclusion, which in turn increases the risk of depression [19,20]. Moreover, excessive engagement in online games may disrupt daily functioning [21,22]. Internet use is, thus, a double-edged sword that can enhance or compromise mental health.

PIU seems to have consequences also for the weight status of the subjects. Longitudinal studies have shown that there is an increased body mass index (BMI) among teenagers who spend many hours daily online [23] and that Internet use significantly increases the odds of being overweight and obese with troubling consequences for health, since it is well known that obesity is a critical risk factor for cardiovascular diseases. Shen et al. found an association between high use of digital technology, including Internet use on mobile phones and video gaming, with higher BMI z-scores and greater odds of being overweight in adolescents [24]. The associations were consistent across sex, ethnicity, and socioeconomic status. However, this association is not always confirmed [25] and needs further investigation. In particular, more comprehensive measurements of other obesity-related lifestyle factors, such as physical activity (PA) and sports practice, are important to unravel potential biological and behavioral mechanisms linking digital technology use and adolescent obesity.

Adolescent behavior is guided by the environment and socialization [26]. In this regard, positive protection is given from PA practice. PA and sports practice have several positive effects on overall physical and mental health and lifestyle habits [27]. Studies have demonstrated that PA practice in adolescents is associated with lower PIU [28,29,30]: PIU is less prevalent among adolescents who practice sports or non-regulated PA (11.5%) or practice federated sports in a club (7.7%) compared to those who do not practice sports (19.2%) [29]. Costigan et al. indicated a small negative association between sedentary screen time and the practice of PA [31], and Villanueva-Blasco et al. reported that adolescents who practice more PA and with a greater intensity did not show PIU [28]. This fact highlights the need to promote PA and sports as a strategy to prevent sedentary lifestyles associated with screens. However, the results regarding the effect of PA or sports practice on PIU are lacking and still contradictory, since some authors found a lack of significant relationship [32] or indicated that PA is an independent behavior, unrelated to other health behaviors [33] or reported that the higher the level of PA, the less likely PIU will occur [34,35], and that individuals who do not practice sport regularly have higher addiction scores than those who do [36,37]. Lapousis and Petsiou reported that the use of the Internet is independent from the participation of individuals in exercises and PA [38]. Differences were also not found by these authors among students with different scores, from moderate to outstanding in relation to their involvement in sports groups, sports clubs, or gyms, nor with exercise training frequency per week. Thus, more studies are needed for a more extensive investigation of the relationship between PA/sport practice and PIU in adolescence.

Considering that the period of health emergence has accentuated sedentary behavior among adolescents and that adolescence represents a very delicate period of life, it is necessary to identify protective factors to prevent the development of PIU [39].

As already mentioned, in the scientific literature, it is still unclear whether sports practice can alleviate or reduce the symptoms of Internet use in adolescents. In other words, if there is some association between PA and Internet use, whether the probability of PIU can be reduced by sports practice will be a very meaningful topic. Moreover, given the importance of the socio-cultural context, few studies regarding PIU focused on the Italian population [39,40,41] and none of them investigated the relationship between PIU and structured PA. Thus, due to the uncertain results of the literature, we carried out this pilot study.

In particular, in this pilot study, we decided to consider only adolescent volleyball players as a sportive group, so that the frequency and intensity of PA were homogenized. Volleyball is one of the most popular team games. More than 200 million people of all ages practice it worldwide [42], and among them many are adolescents. In this sport, the training and competition load consists of alternating low- and high-intensity actions [43]. In addition, because the data collection of this study was based on online questionnaires due to COVID-19 restrictions, the physical practice in the chosen sample of sports adolescents can be simply certified by sports clubs. This study aimed to analyze the Internet use in adolescent volleyball players in comparison with nonsportive peers to verify whether (1) sport practice is associated with non-problematic Internet use, (2) there are different attitudes between sexes and (3) there is an association between BMI, sport practice and Internet use. We hypothesized that sportive adolescents show less PIU than nonsportive ones as well as males less than females, and that BMI has a positive association with PIU.

## 2. Materials and Methods

### 2.1. Participants

The study was carried out between December 2020 and March 2021 on a convenient sample of 142 Italian adolescents from North Italy, subdivided into two groups, sportive and nonsportive (non-participating in any sport).

The sportive group was composed of 66 volleyball players: 37 males (mean age: 15.6 ± 1.1 years) practicing volleyball at the junior teams of the Treviso Volley sports club and 29 females (mean age: 15.6 ± 1.1 years) practicing volleyball at the junior teams of the Albatros Volleyball sports club. Both clubs are based in Treviso (Northern Italy). All players trained for 10 h/week. Inclusion criteria were the following: a 12–18-year age range, practicing volleyball, and being Italian. All the volleyball players of the junior teams of the two volleyball clubs participated in the study.

The nonsportive group was recruited via social media by contacting students of a high school in Novara (Northern Italy): of the 212 students who voluntarily joined (26.6% of adhesion), 76 reported that they had not played any sport regularly in the past two years, and they were included in our comparison sample (nonsportive group). This group was composed of 17 males (mean age: 16.2 ± 1.1 years) and 59 females (mean age: 16.0 ± 1.2 years). Inclusion criteria were the following: age range between 12 and 18; not practicing any sport, and being Italian. All participants were invited to fill out an online retrospective questionnaire that covered Internet use during the COVID-19 lockdown, in addition to the key demographic (gender and age) and anthropometric characteristics. Participation in the study was entirely voluntary and anonymous. After the participants were adequately informed of the purpose of the research, written informed consent was obtained from the parents or legal guardians of all adolescents included in the study. The study was approved by the Bioethics Committee of the University of Bologna (Ethical Approval No. 2.18).

**Figure 1:**
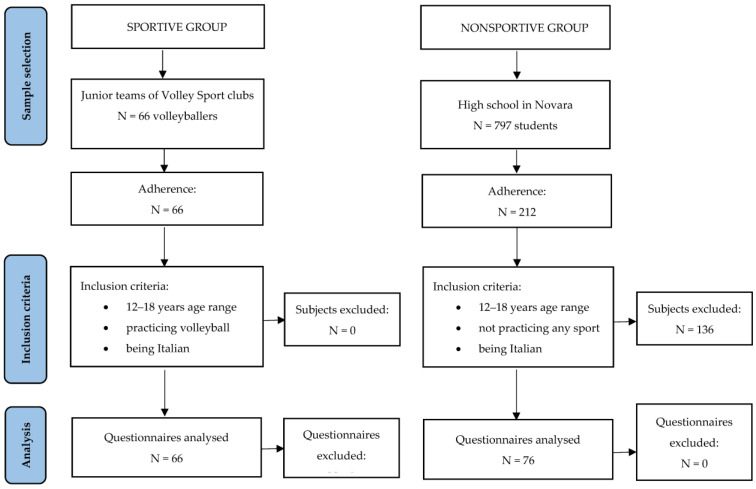
Flow diagram for the study outline, including sample selection, size, and criteria for inclusion.

Figure 1 displays the outline of the study.

### 2.2. Procedures

Anthropometric characteristics (standing height and weight) were self-reported by each participant, and body mass index (BMI) was calculated (weight (kg)/height squared (m^2^)). Each subject was thus classified into underweight, normal weight, overweight, or obese groups according to the BMI Cole et al. cutoff values by sex and age [44,45].

Internet addiction or PIU was assessed using the Use, Abuse, and Dependence on Internet (UADI) inventory, administered online to the subjects using the platform Google Forms [46]. The validated questionnaire is composed of 75 items, each with 5 possible answers according to a Likert scale: “absolutely false” (1), “rather false” (2), “neither true nor false” (3), “quite true” (4), and “absolutely true” (5). It identifies five dimensions: Compensatory Escape, Dissociation, Real Life Impact, Experience Making, and Addiction. Compensatory Escape measures the extent to which individuals use the Internet to avoid daily difficulties, compensating for these difficulties through mood regulation, personal self-efficacy, and online social relationships. Dissociation describes the presence of alienation and dissociative symptoms. Real Life Impact measures the consequences of Internet use on daily life. Experience Making concerns the extent to which individuals use the Internet as a tool to experiment with the self. Finally, Addiction analyses symptoms of dependence, including tolerance, withdrawal, and compulsiveness. Higher scores on all five dimensions indicate more severe addictive Internet behaviors. An adequate internal consistency for the five cited dimensions was detected in the study of Del Miglio et al. [46] (0.76 < α < 0.93).

Following the subsequent proposal of Cantelmi et al., the total raw score of each scale was then standardized in the T-score, which concerns a normal distribution with a mean of 50 and standard deviation of 10 to allow for the immediate classification of the subject [47]. According to this new scale, subjects with a score between 31 and 69 are classified as “normal”, those who exceed the score of 70 (that is who differ by two standard deviations from the mean) are considered “pathological”, and a score of less than 30 identifies a subject “with a psychological condition very far from Internet addiction”.

### 2.3. Statistical Analysis

Variable normality was verified with the Kolmogorov–Smirnov test. Descriptive statistics (means and SD) were calculated for quantitative variables, and the percentage frequency was determined for qualitative variables.

Differences in the frequencies were tested by the chi-squared test. An independent t-test was carried out to evaluate the differences in anthropometric measurements between sportive and nonsportive individuals within the same sex. Two-way ANOVAs adjusted for age were performed on all the questionnaire items, total score, z-score, and the T-score of five dimensions of the UADI inventory to test the differences between sexes, sports groups, and their interaction (group*sex). When a significant F ratio was obtained, the Tukey post hoc test was used to evaluate the differences among groups. Effect sizes using partial eta squared were calculated and interpreted using the benchmarks provided by Cohen (0.01 = small, 0.06 = medium, and 0.14 = large) [48]. Correlations between the T-score of each dimension and BMI were performed separately for the four subsamples and finally for sport groups and sex.

The data analysis was performed using Statistica for Windows version 8.0 (Stat Soft, Tulsa, OK, USA), and the significance level for all statistical tests was set at *p* < 0.05.

## 3. Results

Table 1 shows the total score, the z-score, and the T-score for all five dimensions of the UADI inventory, and in Figure S1, the mean T-scores for each sex and sport groups are reported. The first dimension is related to Compensatory Escape. The mean T-scores of all the groups fall within the “normal” category, indicating that the samples did not use the Net as an escape from real problems or did not have difficulty in establishing social relationships. When analyzing individual data, only five subjects (three nonsportive females, one sportive female, and one sportive male) exceeded the threshold of 70. If the single items were considered (Table S1), more differences emerged between sexes than between groups by sports practice, even if the latter were also noteworthy. In regard to sex differences, females in the nonsportive group generally had higher mean values than nonsportive males, while the differences were not uniform in the sportive groups.

For the second dimension (Dissociation), the mean values of the groups denoted an absence of depersonalization or escape from reality. The same five people who exceeded the threshold of 70 in Compensatory Escape also exceeded the threshold for 70 in Dissociation. Both sportive and nonsportive females had higher mean values than their male peers. Sportive males generally presented higher mean values than nonsportive males, whereas in females the results were heterogeneous.

Regarding Real Life Impact, the values of six subjects exceeded the threshold of 70: three nonsportive females, one nonsportive male, and two sportive males. Both groups and sexes had a significant impact on the values of the single items. Nonsportive groups of both sexes showed higher mean values than sportive groups, while sportive males presented higher values than sportive females in almost all items. For nonsportive subgroups, the values of the single items were not consistent between the sexes.

The mean values for the fourth dimension, Experience Making, did not show particular imbalances related to the presence of transgressive or aggressive behaviors or lying attitudes. It emerged as a natural need to express oneself in new roles or identities. Five males (two nonsportive and three sportive boys) and one sportive female exceeded the threshold of 70. In nonsportive groups, males presented higher values than females in the majority of the items, whereas no consistent trend emerged in the other comparisons.

Regarding Addiction, mean values indicated that participants from all groups showed adequate management of the network. Only three females (two nonsportive girls and one sportive girl) showed values higher than 70. Regarding the single items, sportive males and females generally presented higher values than their nonsportive peers. Females had generally higher values than males, with differences in a larger number of items for the nonsportive group.

In the sample, only three subjects exceeded the threshold of 70 in three dimensions (one sportive girl and one sportive boy in the dimensions of Experience Making, Compensatory Escape, and Dissociation, and one nonsportive girl in the dimensions of Compensatory Escape, Dissociation, and Addiction) and two nonsportive girls exceeded the threshold of 70 in two dimensions (Compensatory Escape and Dissociation). Partial eta squared generally indicated a small effect size of the two factors analyzed.

Table 2 shows anthropometric parameters and weight status separately by sex and sports practice. As expected, sportive boys and girls were taller than their nonsportive peers. For BMI, a significant difference was found between sportive and nonsportive males, but the same was not observed in females. Nonsportive males presented significantly higher mean BMI and a higher incidence of nutritional/weight disorders than sportive males (58.8% of overweight/obese in nonsportive vs. 20% in sportive).

Considering the correlation between the T-score of each dimension of the UADI inventory and BMI in sportive and nonsportive participants according to sex, generally, no statistical significance was observed (*p* > 0.05). The only exception regards the sportive females, which show a significant correlation between BMI and the first and second dimension (*p* < 0.05).

## 4. Discussion

The purposes of this study were to evaluate whether sports practice could have a positive association with avoiding Internet addiction by comparing the use of the Internet in an Italian sample of nonsportive adolescents and sportive adolescents practicing volleyball, and to analyze a possible difference in attitude between sexes during the social distancing caused by COVID-19. Moreover, the association between PIU and BMI was investigated.

Regarding the general topic addressed, we believe that the present study provided some remarkable, albeit preliminary, results. While the scientific literature still shows contradictory results regarding the effect of PA on PIU [28,29,31,33,38], this pilot study found that both sports practice and sex presented an association with Internet use, with a more positive attitude generally revealed in sportive adolescents and in males.

**Figure 2:**
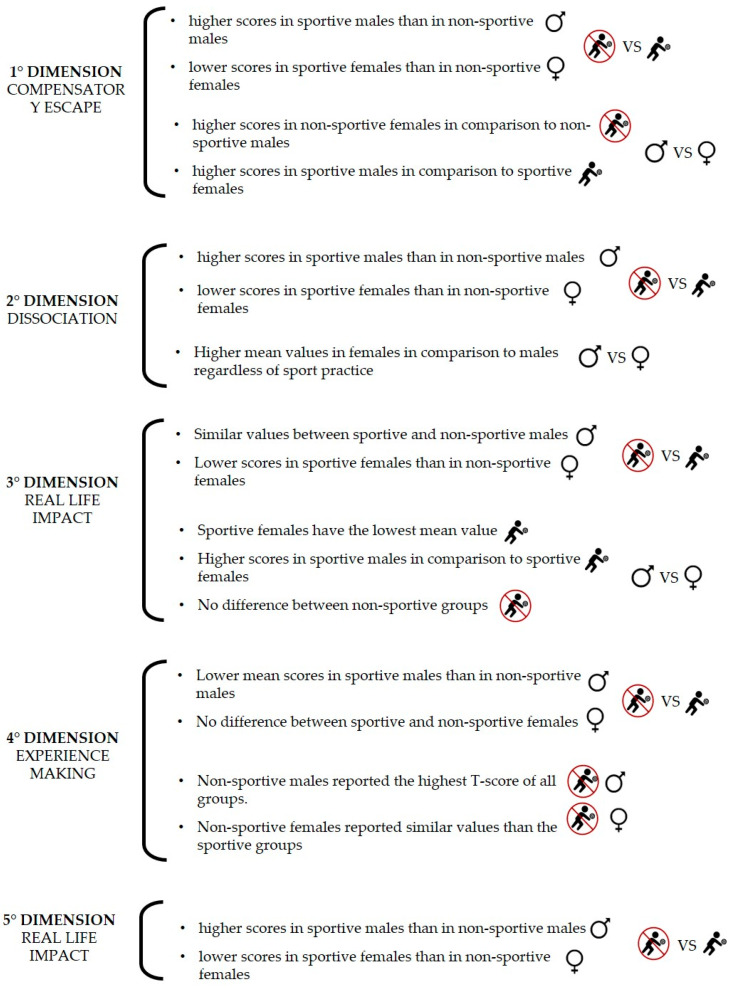
Summary graph reporting the main results of the study.

Through the application of the UADI questionnaire, the resulting T-scores did not suggest, on average, pathological aspects in the adolescents of the present study; in any case, different trends for the five dimensions were observed between sportive/nonsportive groups and sexes (Figure 2); no association was found with the results of the UADI questionnaire and BMI.

Beginning with the first point, we considered the differences between sportive and nonsportive individuals: the results vary according to the considered domain, making it difficult to establish a clear picture or trend. In regard to the compensatory escape, a different tendency was observed in the two groups: sportive males showed higher values than nonsportive males, and the opposite was observed in females. Thus, the use of the Internet for online contacts seemed preferable over personal contacts in sportive males and nonsportive females. According to Kardefelt-Winther [49], negative life situations can cause the motivation to log in online to alleviate negative feelings and indicate compensatory Internet use. Sportive males and nonsportive females seemed to manifest a greater ease of social interactions via the Internet as well as a greater stimulus and the sense of consolation and escape from difficulties given by the use of the network. Social networks are used in the context of identity development and offer a space to grow, watched by peers, and supported by a group that the teen seeks to feel a part of [50,51,52]. Compensatory Internet use has been often associated with videogame usage, understanding that online activities, such as video gaming, may fulfill individual needs unachieved in real life [53].

For dissociation generally defined as a disruption in the usually integrated functions of consciousness, memory, identity, and perception of the environment [54], sportive males generally presented a higher sense of dissociation than nonsportive males; a superiority in the total score of this domain was detected in nonsportive females in comparison with sportive ones. In general, the subgroups of females reported higher values than those of males, showing a higher tendency towards dissociation from reality.

Nonsportives of both sexes were more affected in real life by the distorted use of the Internet, choosing to stay more on the Internet rather than with their friends or relatives, and having a greater sense that the Internet interferes negatively with their studying or with their social relationships. This difference between groups could be probably due to the social relations induced by sporting practice which led the sportive group to have a more concrete contact with reality. More generally, teenagers are motivated to use social networks because they can develop an identity based on an idealized profile; this profile must be at least as good as those of their peers to obtain their approval, and this aspect seems to be more pronounced in nonsportive adolescents [51].

Sportive and nonsportive groups did not show evident differences for the fourth dimension, Experience Making.

Contrary to expectations, addiction, which includes tolerance (increase the progressive number of the connection period), abstinence, compulsivity, and over-involvement values were higher in sportive males than their nonsportive peers.

Few studies reported a significant effect of PA on IA: the higher the level of PA, the less likely IA is to occur [15,34,35]. According to Kabadai, the students who were regularly engaged in sports activities had lower addiction levels when compared with students who were not [36]. This suggests that PA and sport practice, as an economic and effective measure to improve IA, have a preventive and curative effect on IA. PA is not only effective in improving IA, but also helps to promote physical and psychological health, increased willpower, and more self-discipline. In [55,56], a significant effect of sports participation on Internet addiction was reported, mediated by self-control; this suggested that, especially for adolescents, PA can be used to prevent and lessen IA by modifying and adopting self-control development programs. In agreement with this statement, Wang et al. reported that physical exercise can not only negatively affect the IA of college students directly, but also indirectly affect the IA through part mediating effects of loneliness and learning burnout, respectively [57].

Moreover, several research studies highlighted the increased risk of PIU during the COVID-19 pandemic, as technology has been used to reduce stress and anxiety [58,59,60]. Similar results were also found in Italian students [61]. Participation and PA practice have been suggested as strategies to increase health benefits and decrease the risk of PIU, especially during lockdowns [59].

The second aspect that was considered was gender differences. Females tended to show higher mean values than males, except for Real Life impact and Experience Making. For Compensatory Escape, differences between sexes were more evident in the nonsportive group, with females presenting higher values, while in the sportive group, a variegate trend was observed with a tendency to show higher scores in males in comparison to females. Regarding this aspect, the literature shows contrasting results. Di Nicola et al., in their study on Italian adolescents, did not find gender differences for PIU, while problematic gambling was significantly more common in males [62]. Escapism through the Internet may constitute a coping strategy to face adversity, which in some cases can be maladaptive. This process is involved in a wide range of psychological problems, consisting of taking measures to influence or avoid negative experiences as much as possible to regulate emotions. Female gamers showed higher escapism scores associated with more problematic gaming [59]. Females showed a higher tendency to escape and dissociate from reality than males, feeling far from reality when online or having difficulties reducing the time of Internet access. This is in accordance with the results of Deleuze et al., who reported that females had a higher preference for virtual environments than males [53]. It is likely that females are more attracted by social motivations and exploration, while males are more competition-oriented [63,64].

Females of both groups had higher values of dissociation than males consistently with a previous study on a sample of Italian adolescents aged between 14 and 18 characterized by a marked gender trend in dissociation and higher scores in the sample of females [39]. Since the loss of time during the connection when teenagers are emotionally involved strengthens the dissociative phenomena related to addiction [65], dissociation represents a potential risk in cases of the abuse of navigation systems, especially for adolescents and their identity in construction. However, non-pathological dissociative symptoms are quite frequent in the population, and dissociation cannot be viewed simply as a learned strategy to reduce emotional involvement [66] but rather as a structural emotion, a sometimes dysfunctional, regulatory strategy [67,68]. Considered together, the propensity to experience dissociative states, the inability to express and understand self-affects, the tendency to act quickly and without reflective thoughts, together with a reduced ability to experience pleasure in daily life, can therefore be considered vulnerability factors for addictive behaviors in adolescence [62], and the girls of the present study seem to be more vulnerable.

A clear difference in the Real Life impact was observed between sexes in the sportive group, where males seem to present a higher necessity to stay on the Internet rather than with their friends or relatives than females. In the nonsportive group, the superiority of the answers alternated in boys and girls unevenly.

Analyzing the answers to the items for the fourth dimension, Experience Making, it appears that nonsportive males tend to more frequently conceal the real purpose of a prolonged connection, in some cases hiding their own identity, using aggressive language, looking for erotic material, or talking about sex. In the sportive group, sex differences were less definite for this dimension. According to Alexandraki et al., 53% of American boys and 28% of girls view pornography, mostly on the Internet; pornography offers an escape [69] from everyday frustrations, offering a sense of omnipotence [69,70]. Chao and Yu [71] showed that male students assigned more importance to the attitude toward Internet-related enjoyment dimension than female students, and males had increased online gaming and pornography viewing times compared to girls. In addition, the authors found that excessive use of online games and cyber pornography could make users lose themselves in the virtual world; it can also affect their self-consciousness. Behavior differs between the two sexes: male adolescents tend to play online games, and female adolescents view social interactions (emailing friends) and searching for information as more important [72]. In essence, then, male gamers begin playing video games early, play more frequently, and spend more time gaming than females [73].

In our study, the addiction was more marked in females than in males, with differences in a larger number of items for the nonsportive group; nonsportive girls found more difficulties disconnecting from the Internet and, in particular, three participants (2.1% of the total sample) with Internet addiction were among the female group. Contrasting results were obtained in the study of Vigna-Taglianti and colleagues on a sample of Italian students: 12.1% were classified as having maladaptive use (14.2% males and 10.1% females) and 0.4% as having Internet addiction [74]. These differences could be due to the fact that the data collection for the cited study was carried out 10 years before ours, and Internet use evolved and increased over time [75], especially during the period of the COVID-19-induced lockdown. While during the years of 2010–2011, the percentage of Internet users was 29–30% of the global population, by 2022–2021, it more than doubled to 64–66% [76]. Internet addiction in adolescence can have a negative impact on identity formation [77] and change the structure of the developing brain [78,79], as it may negatively affect cognitive functioning, leading to poor academic performance and engagement in risky activities, poor dietary habits, low-quality interpersonal relations, and self-injurious behavior [80,81,82,83].

In regard to weight status, it should also be mentioned that sportive males presented better values of BMI (significantly lower in males) and lower weight disorder values than nonsportive ones. Females, on the other hand, did not show significant differences between groups, although the presence of overweight and obese adolescents was higher in the nonsportive group. Sex differences could be connected to the diversity in weight-related concerns among the two sexes, since girls, regardless of sports practice, generally report higher levels of weight-related concerns compared with boys, including the desire to lose weight and body image dissatisfaction [84,85]. Furthermore, the lower female ecosensitivity than male one contributed to the observed pattern concerning the two different environments (sportive and nonsportive) [86]. In any case, in the present study, generally, no significant correlations were found between the results of the five domains and the BMI. Only sportive females showed a correlation between BMI, Compensatory Escape and Real Life impact. Thus, as BMI increased, sportive girls showed a greater need to use the Internet as compensation and showed greater changes in social relationships and mood due to Internet use. The association between PIU and weight status is not so well defined, as reported by the scientific literature [25]. In their review, Aghasi and colleagues [87] reported that Internet use was significantly associated with increased odds of being overweight and obese. Sari and Ayding [88] also reported a significant positive correlation between BMI and PIU in university students, whereas Tsitsika and colleagues and Shen et al. found this correlation in European adolescents [24,89]. On the other hand, Barrense-Dias et al., analyzing 621 Switzerland students from 14 to 16 years old, found no association between BMI and Internet use [90]. These non-consistent results may be due to the cultural differences among samples and to the heterogeneity of questionnaires used to determine PIU and Internet use in general.

### Strengths, Limitations, and Recommendations for Future Research

Some limitations that could affect the generalization of the results should be noted. First of all, convenience sampling, the limited sample size, and the numerical inhomogeneity between males and females in the nonsportive group, as the majority of nonsportive participants were females, should be noted. The only type of sport considered was volleyball. Weight and standing height were self-reported, which may have consequences for the reliability of the results. The forced social distancing imposed by COVID-19 may have influenced the behavior of the subjects. In addition, a limited number of variables were collected: we considered the impact of sports activities on PIU, but we had no information regarding other aspects that could have an impact, too, such as sociodemographic variables, socioeconomic status, leisure activities, etc. Moreover, the comparison with other studies was difficult due to the heterogeneity of the questionnaires used to investigate the PIU. The cultural background of the sample could influence the results. Last, the cross-sectional design of the study did not allow the establishment of causal inferences; therefore, only the association between the variables could be established. Further research should consider these limitations, opting for a longitudinal approach.

The major strength of this study is the use of a validated questionnaire on the Italian population that includes a high number of items allowing the evaluation of various aspects that cover all the features of Internet use. Moreover, this study investigated Internet use in sportive adolescents, a topic poorly represented in the literature.

## 5. Conclusions

In conclusion, the results of the present pilot study highlight a general tendency towards a negative association between sports practice and PIU, especially among females. Regarding the considered factors, gender seems to present higher association than sports practice with PIU: females generally presented a greater predisposition to escape from reality and a greater dependence on Internet use than males. In addition, more females than males scored above the thresholds simultaneously on two or more dimensions. Thus, special attention should be given to girls during adolescence, especially to those that do not practice any sport. Teenagers are likely the most susceptible population segment: they are particularly vulnerable to the initiation of addictive behaviors, and they are the population subgroup most exposed to the PIU. Further longitudinal research is needed to examine the functions and potential long-term effects of the many distinct and rapidly evolving uses of the Internet, especially in the current health emergency due to the spread of COVID-19, which has forced the population to remain isolated for long periods. Adolescents are the ones who can suffer most from these restrictions and the lack of social contact and can take refuge most in the use of the Internet.

Overall, this first pilot study showed promising results. In conducting large-scale research, researchers are urged to take into account the social and developmental context of adolescents’ daily lives.

## Figures and Tables

**Table 1:** Total, z, and T-scores and ANOVA for the five dimensions of the UADI inventory: comparisons between means of sportive and nonsportive groups (“Group”), males and females (“Sex”), interaction between sport and sex (“Group*sex”).

	Males				Females				ANOVA								
	Nonsportive		Sportive		Nonsportive		Sportive		Group			Sex			Group*sex		
Dimension	Mean	SD	Mean	SD	Mean	SD	Mean	SD	F	*p*	Partial Eta^2^	F	*p*	Partial Eta^2^	F	*p*	Partial Eta^2^
**Compensatory Escape**																	
Total	36.1	12.1	39.2	9.5	39.1	10.9	38.2	10.6	7.8	0.005	0.003	7.0	0.008	0.003	16.3	0.000	0.007
z-score	−0.2	1.1	0.0	1.0	0.1	1.0	−0.1	1.1	1.7	0.194	0.001	6.1	0.013	0.003	15.6	0.000	0.007
T-score	48.2	11.1	50.4	9.5	50.9	9.9	49.5	10.7	1.7	0.194	0.001	6.1	0.013	0.003	15.6	0.000	0.007
**Dissociation**																	
Total	30.4	13.1	32.1	8.6	34.9	11.0	35.0	9.3	3.9	0.049	0.001	68.1	0.000	0.029	3.9	0.048	0.002
z-score	−0.3	1.2	−0.1	1.0	0.1	1.0	0.1	1.2	0.5	0.476	0.000	46.3	0.000	0.020	5.3	0.021	0.002
T-score	47.3	11.7	48.6	9.6	51.3	9.9	50.5	12.5	0.5	0.476	0.000	46.3	0.000	0.020	5.3	0.021	0.002
**Real Life Impact**																	
Total	44.7	6.4	40.6	7.2	44.5	7.4	38.7	6.6	13.5	0.000	0.096	10.8	0.001	0.005	5.7	0.017	0.003
z-score	0.1	0.9	0.1	1.0	0.0	1.0	−0.2	0.9	3.1	0.080	0.001	11.1	0.001	0.005	6.0	0.014	0.003
T-score	50.7	8.9	51.2	10.4	50.4	10.3	48.5	9.5	3.1	0.080	0.001	11.1	0.001	0.005	6.0	0.014	0.003
**Experience Making**																	
Total	37.9	10.2	39.0	8.5	36.2	6.6	37.2	7.4	11.0	0.001	0.005	19.4	0.000	0.009	0.3	0.601	0.000
z-score	0.2	1.4	0.1	1.1	0.0	0.9	−0.7	1.8	3.8	0.053	0.022	4.2	0.040	0.034	4.0	0.046	0.014
T-score	52.2	13.5	49.9	9.7	50.0	8.7	50.0	9.6	3.8	0.053	0.002	4.2	0.040	0.002	4.0	0.046	0.014
**Addiction**																	
Total	43.4	8.4	46.5	9.4	46.6	8.6	43.6	18.6	0.0	0.927	0.000	0.1	0.771	0.000	33.6	0.000	0.022
z-score	−0.2	0.9	0.2	0.6	0.1	1.0	−0.1	1.2	3.8	0.050	0.002	1.5	0.218	0.001	50.8	0.000	0.022
T-score	47.7	9.4	51.6	5.8	51.3	9.7	49.0	12.4	3.8	0.050	0.002	1.5	0.218	0.001	50.8	0.000	0.022

SD = standard deviation, *p* = *p*-value.

**Table 2:** Comparison by T-test and chi-squared test of the anthropometric characteristics and weight status by sports practice separately for each sex.

	Males						Females					
	Nonsportive		Sportive				Nonsportive		Sportive			
Anthropometric Variables	Mean	SD	Mean	SD	t	*p*	Mean	SD	Mean	SD	t	*p*
Weight (kg)	76.2	22.5	70.6	10.2	1.3	0.195	58.9	11.4	61.7	9.2	−1.2	0.219
Height (cm)	173.8	10.4	182.2	8.3	−3.2	0.002	163.1	6.4	171.2	7.4	−5.6	0.000
BMI (kg/m^2^)	24.9	5.8	21.2	2.1	3.6	0.001	22.2	3.9	21.0	2.2	1.7	0.100
Weight status (%):					ꭓ^2^	*p*					ꭓ^2^	*p*
Underweight	17.6		2.5		18.7	0.000	5.3		8.6		4.0	0.259
Normal weight	23.5		77.5				71.9		829			
Overweight	41.1		20.0				15.8		8.6			
Obese	17.6		-				7.0		-			

SD = standard deviation, *p* = *p*-value.

## Data Availability

Data are available upon request due to ethical restrictions regarding participant privacy. Data requests may be sent to the corresponding authors.

## Supplementary Materials

The following supporting information can be downloaded at: https://www.mdpi.com/article/10.3390/healthcare11233075/s1, Table S1: Total, Z, and T scores and ANOVA for the single items of the five dimensions of UADI inventory by sex, sport group, and interaction (Group*sex). Figure S1: Histogram representing the mean values of T score for all the five dimensions separately by sex and sport practice.
