# Supplementary material for: A Pilot Study on the Association of Internet Use with Sports Practice and Sex in Italian Adolescents
Source: Healthcare (Basel). 2023 Nov 30;11(23):3075. doi: 10.3390/healthcare11233075 (PMC10706377; doi:10.3390/healthcare11233075)

SUPPLEMENTARY MATERIAL

A pilot study on the association of Internet use with sports practice and sex in Italian adolescents

Table S1. Total, Z and T scores and ANOVA for the single items of the five dimensions of UADI inventory by sex, sport group, and interaction (Group*sex).

	Non-sportive males		Sportive males		Non-sportive females		Sportive females		Group		Sex		Group*sex	
DIMENSION and items	M	SD	M	SD	M	SD	M	SD	F	p	F	p	F	p
Real Life Impact														
59. I do not try to hide from other people the amount of time I spend online.	3.3	1.4	2.4	1.1	2.8	1.3	2.4	1.1	142.4	0.000	18.3	0.000	18.0	0.000
49. I never choose to stay on the Internet rather than with my friends or relatives.	2.5	1.3	1.9	1.1	2.3	1.5	1.7	1.0	103.5	0.000	10.0	0.002	0.4	0.512
43. The Internet does not interfere negatively with my job. my studying or with my social relationships.	3.2	1.1	2.9	1.0	3.3	1.1	3.1	1.3	25.8	0.000	25.8	0.000	2.6	0.105
64. During the day I never miss the Internet.	3.1	1.4	2.7	1.3	2.8	0.9	2.7	1.0	16.9	0.000	4.8	0.028	5.6	0.019
35. I do not feel the sensation to travel. to dream or to be in a movie when I am online.	2.8	1.2	2.8	1.4	3.5	1.3	2.4	1.2	90.2	0.000	10.9	0.001	70.1	0.000
41. When I stay together with my friends or relatives. I never think of the Internet.	2.6	15	2.7	1.2	2.7	1.2	2.5	1.0	1.8	0.183	2.9	0.090	3.2	0.076
70. I do not consider online relationships more satisfying than the actual ones.	2.2	1.6	1.8	1.2	2.5	1.4	1.7	1.0	105.0	0.000	1.1	0.284	7.0	0.008
22. My friends or relatives do not complain because I spend too much time online.	2.9	1.0	3.6	1.2	3.3	1.2	3.7	1.3	78.0	0.000	10.1	0.002	12.5	0.000
39. I am not embarrassed or reserved when someone asks me what I do online.	2.8	1.3	3.1	3.4	2.7	1.2	2.5	1.2	0.4	0.549	12.0	0.001	9.6	0.002
14. Since I have been using the Internet. I have never skipped a meal or modify my habits.	2.8	1.4	2.2	1.5	2.8	1.4	1.9	1.1	145.3	0.000	8.1	0.004	9.8	0.002
32. The Internet never influences my mood.	3.4	1.3	2.9	1.1	3.3	1.2	2.9	1.2	76.3	0.000	1.9	0.168	0.1	0.711

73. Online I do not care about other people's opinion about me	3.5	1.3	2.9	1.4	3.3	1.3	2.7	1.2	102.9	0.000	6.2	0.013	0.4	0.552
66. I do not feel (physically or psychologically) tired when I use the Internet.	2.7	1.3	2.3	1.3	2.8	1.3	2.7	1.3	19.9	0.000	19.0	0.000	1.8	0.179
51. I do not think that social relations are less stressful online.	3.4	1.1	3.0	1.2	3.2	1.2	2.8	1.0	51.0	0.000	16.4	0.000	0.5	0.462
27. I never feel strong emotions on the Internet.	3.5	1.2	3.3	1.3	3.1	1.1	3.2	1.4	1.6	0.212	26.6	0.000	7.2	0.007
**TOTAL DIMENSION**	44.7	6.4	40.6	7.2	44.5	7.4	38.7	6.6	13.5	0.000	10.8	0.001	5.7	0.017
**Z score**	0.1	0.9	0.1	1.0	0.0	1.0	-0.2	0.9	3.1	0.080	11.1	0.001	6.0	0.014
**T1 score**	50.7	8.9	51.2	10.4	50.4	10.3	48.5	9.5	3.1	0.080	11.1	0.001	6.0	0.014
Experience Making	M	SD	M	SD	M	SD	M	SD	F	p	F	p	F	p
7. When I am on the Net I do not hide my true identity.	3.0	1.3	2.5	1.3	2.6	1.5	2.5	1.4	21.4	0.000	12.4	0.000	7.4	0.007
18. Sometimes I find it fun to lie on the web.	2.3	1.3	2.3	1.0	1.9	1.3	1.8	1.0	0.7	0.417	56.2	0.000	0.8	0.373
13. I use bad or aggressive language on the Internet.	1.9	1.1	1.9	1.0	1.8	1.1	1.5	0.8	12.4	0.000	23.3	0.000	14.9	0.000
15. I prefer to go online when I am lonely or nobody is watching me.	2.2	1.2	2.4	1.2	2.4	1.3	2.9	1.3	40.8	0.000	32.9	0.000	7.5	0.006
25. Online I tend to behave in a different way from usual.	2.4	1.4	2.3	1.2	2.0	1.1	1.9	0.9	1.3	0.247	56.4	0.000	2.6	0.110
30. Online I find myself looking for erotic material or talking about sex.	2.9	1.3	2.9	1.3	1.9	1.2	1.6	0.9	5.6	0.018	469.1	0.000	4.5	0.035
31. I tend to describe myself differently than I am when using chat. messaging apps. email. role playing. dating apps or sites etc.	1.9	1.1	1.8	1.1	1.7	1.0	1.7	0.9	1.6	0.201	11.5	0.001	0.3	0.591
36. Often I go online to dismiss boredom.	3.5	1.4	4.3	0.8	4.0	1.0	4.3	0.8	120.4	0.000	25.0	0.000	23.1	0.000
37. I feel that online my (sexual. social or professional) identity is more fluid and less constrained.	2.4	1.4	2.5	1.2	2.5	1.0	2.8	1.1	23.0	0.000	13.6	0.000	1.2	0.272
9. The Internet is synonymous with transgression.	1.8	1.0	2.0	0.9	2.0	1.0	2.3	1.0	26.4	0.000	24.2	0.000	0.3	0.613
19. I happen to connect if I don't have a specific purpose.	3.2	1.5	3.6	1.1	3.5	1.0	3.9	0.9	81.5	0.000	30.3	0.000	0.2	0.648
42. I think through the Internet. instant messaging. chat. social networking and etc. it is easier to have exciting experiences.	2.6	1.1	2.5	1.1	2.0	1.1	1.8	1.0	9.7	0.002	176.6	0.000	1.8	0.183
60. I think I spend too much time online.	3.3	1.1	3.3	1.3	3.2	1.3	3.6	1.2	11.2	0.001	1.9	0.171	7.3	0.007
65. Often I go online to get my own private space.	2.5	1.3	2.9	1.3	3.0	1.2	3.2	1.2	29.9	0.000	46.4	0.000	4.0	0.045

68. On the Net it can be exciting to change your identity (sexual. social or professional).	1.9	1.1	1.7	0.8	1.8	1.1	1.6	0.8	21.9	0.000	3.2	0.073	0.2	0.695
**TOTAL DIMENSION**	37.9	10.2	39.0	8.5	36.2	6.6	37.2	7.4	11.0	0.001	19.4	0.000	0.3	0.601
**Z score**	0.2	1.4	0.1	1.1	0.0	0.9	-0.7	1.8	3.8	0.053	4.2	0.040	4.0	0.046
**T2 score**	52.2	13.5	49.9	9.7	50.0	8.7	50.0	9.6	3.8	0.053	4.2	0.040	4.0	0.046
Compensatory Escape	M	SD	M	SD	M	SD	M	SD	F	p	F	p	F	p
3. I prefer to contact people via the Internet or by text or voice messages. rather than by phone or in person.	2.5	1.4	2.7	1.2	2.4	1.3	2.3	1.1	5.1	0.024	14.3	0.000	4.6	0.032
4. I get excited about surfing or communicating on the Internet via pc or smartphone or other devices.	1.8	1.0	2.2	1.0	2.2	1.0	1.9	1.0	0.2	0.639	2.2	0.138	51.5	0.000
5. I have the impression that everything is easier on the Net.	2.9	1.1	3.2	1.0	2.9	1.2	3.0	1.1	18.4	0.000	0.6	0.439	3.6	0.059
6. Sometimes I think of the Internet to distract myself from unpleasant thoughts.	3.1	1.3	3.2	1.4	3.4	1.3	3.3	1.3	0.3	0.574	11.5	0.001	1.8	0.179
11. The Internet facilitates my social relationships.	2.5	0.9	3.1	1.2	2.9	1.2	2.9	1.3	47.9	0.000	2.9	0.088	30.9	0.000
20. I happen to have "daydreams" on the Internet.	2.4	1.5	2.2	1.4	2.3	1.3	2.5	1.2	2.3	0.126	4.8	0.029	11.0	0.001
21. The Internet stimulates my imagination without limits.	2.5	1.3	3.2	1.2	2.5	1.3	2.7	1.1	71.1	0.000	17.9	0.000	16.2	0.000
17. I often find myself thinking about when I can come back online next time.	1.9	1.2	2.3	1.3	2.2	1.2	2.1	1.1	9.1	0.003	2.6	0.105	26.3	0.000
57. Sometimes. after a bad day. I need to go online.	2.2	1.4	2.4	1.2	2.8	1.3	2.9	1.6	4.5	0.034	95.3	0.000	0.1	0.745
58. Sometimes the Internet represent for me a way to give vent to my worries.	2.8	1.4	2.8	1.3	3.0	1.3	3.2	1.3	3.2	0.073	25.4	0.000	2.1	0.151
61. I happen to think about what is happening or what I could do on the Net when I am not connected.	2.4	1.4	2.6	1.2	2.3	1.1	2.2	1.4	0.3	0.559	14.5	0.000	4.9	0.027
53. Sometimes I think real life is more depressing than online life.	2.4	1.5	2.2	1.1	2.6	1.2	2.2	1.2	20.2	0.000	5.9	0.015	12.1	0.001
71. Often my mood gets better when I am online.	2.4	1.4	2.5	1.2	2.8	1.2	2.6	1.0	2.3	0.128	36.8	0.000	15.0	0.000
72. Sometimes the Internet lets me feel more important.	2.0	1.4	2.2	1.2	2.5	1.2	2.3	1.1	0.1	0.717	37.6	0.000	8.0	0.005
45. Online I feel more euphoric.	2.4	1.3	2.5	1.1	2.2	1.1	2.1	1.1	0.0	0.931	32.4	0.000	4.2	0.041
**TOTAL DIMENSION**	36.1	12.1	39.2	9.5	39.1	10.9	38.2	10.6	7.8	0.005	7.0	0.008	16.3	0.000
**Z score**	-0.2	1.1	0.0	1.0	0.1	1.0	-0.1	1.1	1.7	0.194	6.1	0.013	15.6	0.000
**T3 score**	48.2	11.1	50.4	9.5	50.9	9.9	49.5	10.7	1.7	0.194	6.1	0.013	15.6	0.000

Dissociation	M	SD	M	SD	M	SD	M	SD	F	p	F	p	F	p
12. Sometimes when I am on the Internet. I feel far from reality or like being elsewhere.	2.1	1.2	2.8	1.3	2.9	1.4	3.2	1.3	55.0	0.000	93.2	0.000	9.2	0.002
23. The Internet causes me a sense of alienation.	1.6	0.9	1.7	0.8	2.2	1.1	2.5	1.1	26.1	0.000	234.9	0.000	4.4	0.036
24. Sometimes I experience flashbacks or incoerent thoughts during or after being on the Internet for a long time.	2.2	1.3	1.8	1.0	2.3	1.2	2.2	1.1	13.3	0.000	20.7	0.000	2.8	0.093
26. I have often tried to reduce the time or frequency of Internet access without success.	2.1	1.2	2.6	1.1	2.5	1.2	2.8	1.1	64.8	0.000	29.2	0.000	10.6	0.001
29. I go online even if I have more important things to do.	3.3	1.5	2.8	1.3	3.0	1.3	2.7	1.3	44.6	0.000	4.1	0.043	5.4	0.020
33. Because of the Internet. I tend to avoid friends or family.	1.6	1.0	1.6	1.1	2.0	1.2	1.8	1.0	1.2	0.277	25.6	0.000	8.2	0.004
38. After a few hours of being connected. people or things around me seem somewhat different.	1.7	1.0	1.8	1.1	2.3	1.3	1.9	1.0	4.9	0.026	34.9	0.000	22.1	0.000
40. I think the Internet is my refuge.	2.5	1.4	1.9	1.2	2.3	1.2	1.9	1.1	75.0	0.000	1.1	0.300	4.0	0.045
44. After a few hours of connection I feel more nervous or more depressed.	2.1	1.4	2.0	1.0	2.3	1.3	2.4	1.0	0.2	0.650	39.9	0.000	2.4	0.120
50. After a few hours of connection I have the feeling that the world around me has something unreal.	1.6	0.9	1.8	1.1	2.2	1.2	1.8	1.0	1.3	0.251	40.6	0.000	30.0	0.000
54. When I am online I feel a vague feeling of omnipotence.	2.2	1.4	2.0	1.0	1.8	1.0	1.9	1.0	1.5	0.227	17.0	0.000	2.9	0.090
55. On the Internet I feel more skilled or shrewd.	2.1	1.1	2.6	1.2	2.1	1.0	2.3	1.0	45.5	0.000	5.2	0.023	6.0	0.015
62. After a few hours of connection. I feel slightly dizzy or have strange sensations.	1.8	1.1	2.4	1.4	2.4	1.3	2.9	1.4	98.9	0.000	82.7	0.000	1.3	0.258
69. Sometimes I feel lost in cyberspace.	1.8	1.1	2.1	1.3	2.3	1.1	2.1	1.1	0.1	0.764	27.4	0.000	17.0	0.000
74. The Internet influences my thoughts or my dreams.	1.8	1.2	2.2	1.1	2.4	1.3	2.6	1.1	19.8	0.000	90.8	0.000	3.7	0.056
**TOTAL Dimension**	30.4	13.1	32.1	8.6	34.9	11.0	35.0	9.3	3.9	0.049	68.1	0.000	3.9	0.048
**Z score**	-0.3	1.2	-0.1	1.0	0.1	1.0	0.1	1.2	0.5	0.476	46.3	0.000	5.3	0.021
**T4 score**	47.3	11.7	48.6	9.6	51.3	9.9	50.5	12.5	0.5	0.476	46.3	0.000	5.3	0.021
Addiction	M	SD	M	SD	M	SD	M	SD	F	p	F	p	F	p
46. I don't get nervous if, for some reason, I can't connect.	3.1	1.5	3.0	1.2	3.0	1.1	2.6	1.4	15.0	0.000	20.8	0.000	4.8	0.028

56. I don't lose hours of sleep because of the Internet.	2.4	1.4	2.7	1.5	3.0	1.2	2.9	1.3	2.9	0.090	53.7	0.000	10.2	0.001
1. I check my email and / or instant messages and / or notifications from apps and social networks at regular intervals.	3.1	1.0	3.5	1.0	3.4	1.0	3.9	1.2	80.8	0.000	51.5	0.000	0.3	0.580
2. When I am on the Net I have the feeling that time flies.	3.5	1.5	4.0	0.8	3.8	1.1	3.9	1.2	38.8	0.000	5.5	0.019	15.3	0.000
8. I get in a bad mood if I have technical connection problems (slow connection or lack of WiFi, etc.).	2.7	1.5	3.0	1.3	3.0	1.3	2.4	1.2	3.3	0.069	4.1	0.042	45.4	0.000
10. It seems to me that the Net is a kind of parallel world.	2.7	1.0	2.9	1.0	3.1	1.0	3.0	1.2	1.4	0.229	34.4	0.000	8.5	0.004
16. Sometimes I stay online more than what was my intention.	3.2	0.9	3.8	1.1	3.6	1.2	4.0	1.1	94.4	0.000	34.5	0.000	7.0	0.008
28. I often do things better thanks to the Internet.	2.9	1.2	3.2	1.1	3.2	1.1	3.3	0.9	28.7	0.000	20.7	0.000	2.4	0.120
34. Sometimes I need to go online, even just for a short time.	2.9	1.5	2.8	1.4	2.9	1.2	2.9	1.3	0.1	0.797	2.0	0.160	0.8	0.382
47. I am disappointed if I do not receive instant messages, notifications from apps, notifications from social networks or e-mails.	2.1	1.2	2.6	1.2	2.4	1.3	2.4	1.2	30.9	0.000	0.8	0.374	22.7	0.000
48. I often check my smartphone, my sites, chats, newsgroups, instant messaging apps, etc.	3.2	1.1	3.6	1.2	3.2	1.2	3.5	1.2	34.0	0.000	1.3	0.260	0.0	0.870
52. I always find a reason to stay online longer.	2.5	1.2	2.9	1.1	3.1	1.2	3.3	1.1	43.1	0.000	89.8	0.000	1.6	0.200
63. It is difficult for me to disconnect from the Internet.	2.4	1.5	2.4	1.1	2.4	1.2	2.6	1.1	1.7	0.198	8.2	0.004	3.6	0.058
67. Sometimes I take comfort using my computer by myself.	3.2	1.2	3.0	1.0	3.0	1.3	3.1	1.4	0.1	0.805	0.1	0.740	7.8	0.005
75. Sometimes I say: "a little bit more ... then I will get off the Internet".	3.5	0.9	3.0	1.0	3.4	1.2	4.2	5.7	1.8	0.183	21.5	0.000	27.5	0.000
**TOTAL DIMENSION**	43.4	8.4	46.5	9.4	46.6	8.6	43.6	18.6	0.0	0.927	0.1	0.771	33.6	0.000
**Z score**	-0.2	0.9	0.2	0.6	0.1	1.0	-0.1	1.2	3.8	0.050	1.5	0.218	50.8	0.000
**T5 score**	47.7	9.4	51.6	5.8	51.3	9.7	49.0	12.4	3.8	0.050	1.5	0.218	50.8	0.000

Note: M=mean, SD=standard deviation, p=p-value.

Figure S1 Histogram representing the mean values of T score for all the five dimensions separately by sex and sport practice

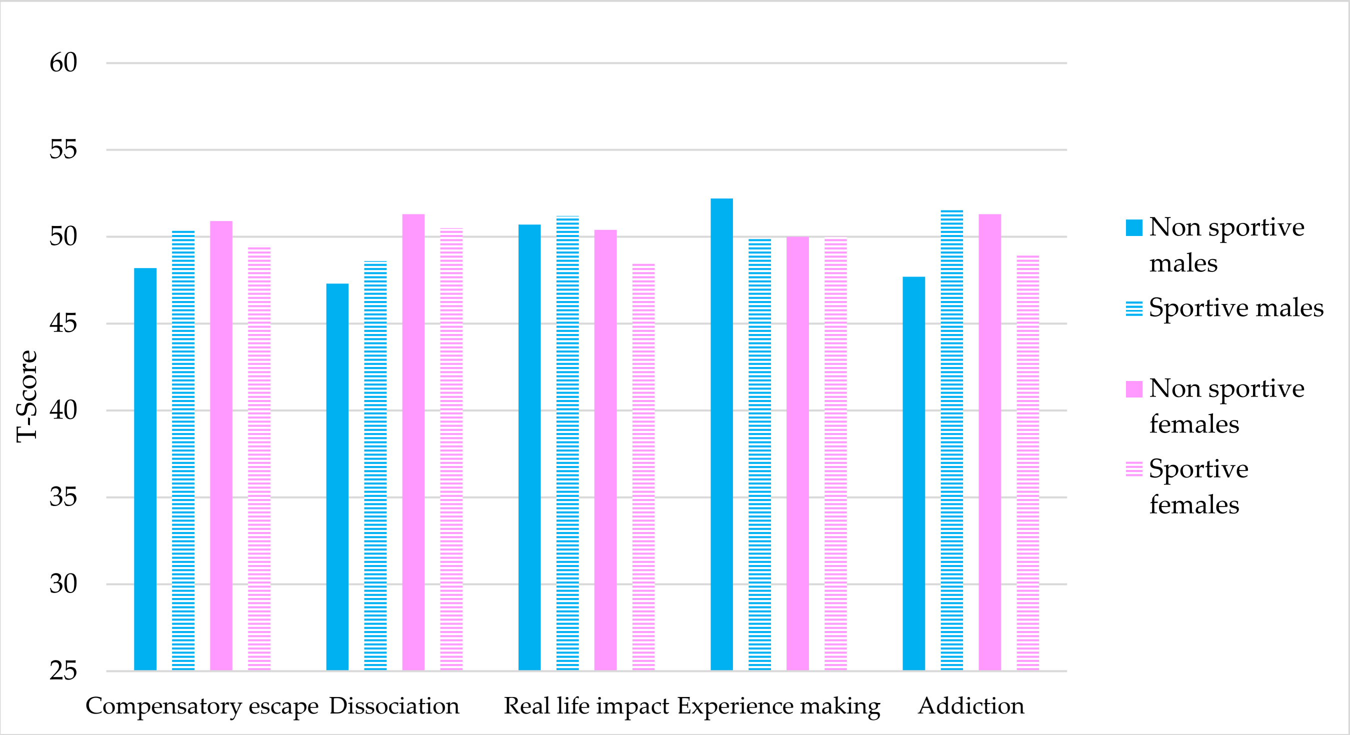

Supplement: Supplementary file 1 [file healthcare-11-03075-s001.zip › healthcare-2703199-supplementary.pdf]
